# Antioxidant Activity of Coconut (*Cocos nucifera* L.) Protein Fractions

**DOI:** 10.3390/molecules23030707

**Published:** 2018-03-20

**Authors:** Yan Li, Yajun Zheng, Yufeng Zhang, Jianguo Xu, Gang Gao

**Affiliations:** 1 Food Science Institute of Shanxi Normal University, Linfen 041004, China; zhengyajun2007@163.com (Y.L.); zhengyajun2017@163.com (J.X.); zhengyajun200705@163.com (G.G.); 2 Coconut Research Institute of Chinese Academy of Tropical Agriculture Sciences, Wenchang 571339, China; zyjcoconut@yeah.net

**Keywords:** coconut cake protein fractions, nutritional value, antioxidant activity, DNA damage

## Abstract

Coconut cake is an abundant and good potential edible protein source. However, until now it has not been extensively used in the food industry. To promote its usage, the characterization, nutrition value and antioxidant activity of coconut cake protein fractions (albumin, globulin, prolamine, glutelin-1 and glutelin-2) were studied. Results revealed that all the albumin, globulin, glutelin-1 and glutelin-2 fractions showed a high nutrition value. The prolamine, glutelin-1 and glutelin-2 all exhibited good radical scavenging activity and reducing power, and the globulin and prolamine showed high ion chelating ability (89.14–80.38%). Moreover, all the fractions except glutelin-2 could effectively protect DNA against oxidative damage. Several peptides containing five to eight amino acids with antioxidant activity were also identified by LC-MS/MS from the globulin and glutelin-2 fractions. The results demonstrated that the coconut cake protein fractions have potential usages in functional foods.

## 1. Introduction

Reactive oxygen species (ROS) and free radicals are very unstable and react rapidly with other groups or substances in the body, leading to cell or tissue injury [1]. Recently, more and more epidemiological evidence has indicated that excessive free radicals and associated oxidative damage are mediators in some chronic diseases, such as diabetes mellitus, neurodegenerative diseases and cardiovascular diseases [2]. In the food industry, oxidation is a major cause of food quality changes that affect the nutritional qualities, the texture and the appearance of the food, leading to undesirable off-flavors and potentially toxic reaction products [3]. Thus, antioxidants play a vital role in both food systems and in the human body to reduce oxidative stress. Since the use of synthetic antioxidants has been suspected to threaten human health, antioxidants from natural sources have attracted more attention. Protein fractions including albumin, globulin, prolamine, glutelin-1 and glutelin-2 are usually classified by solubility [4]. Previous studies have demonstrated that the relative proportion of each fraction in a protein influenced its physicochemical properties, nutritional value, bioactivity and usage in food or other industries [5]. Some protein fractions with antioxidant activity can also be used in food to increase the shelf life of the product. In recent years, protein fractions possessing antioxidant activity have received increasing attention.

Coconut cake, with a global annual output of 200 million tons, is the main byproduct of the coconut milk and oil industry, and contains approximately 10–16% protein [5]. Apart from its nutritional quality, coconut cake protein (CCP) can lower the serum lipid of rats [6]. Albumin and globulin are the major constituents of coconut proteins, accounting for 21 and 40 g/100 g, respectively [4]. Although some physicochemical and functional properties of coconut globulin have been studied, and several antioxidant and antimicrobial peptides have been obtained from coconut water [5,7], there is no information about the nutritional value, functional properties and bioactivity of other CCP fractions (albumin, prolamine, glutelin-1 and glutelin-2). This limits the usage of CCP in the food and medical industries. Therefore, the object of this paper is to characterize the nutritional value and antioxidant activity of coconut cake protein fractions.

## 2. Results and Discussion

### 2.1. Distribution of Protein Fractions

Following sequential fractionation, coconut cake albumin, globulin, prolamine, gultelin-1 and glutelin-2 fractions were separately obtained. Their contents were 8.84 ± 1.16, 54.35 ± 3.69, 0.92 ± 0.30, 20.49 ± 1.11 and 6.14 ± 1.42 g/100 g, respectively. Obviously, globulin was the predominant fraction, in accordance with the report of Angelia et al. [5]. However, the albumin content in CCP was much lower than that in coconut meat protein (21.00 g/100 g) [4], indicating that the methods used in the production of coconut cake caused a loss of albumin.

### 2.2. SDS-PAGE

**Figure 1:**
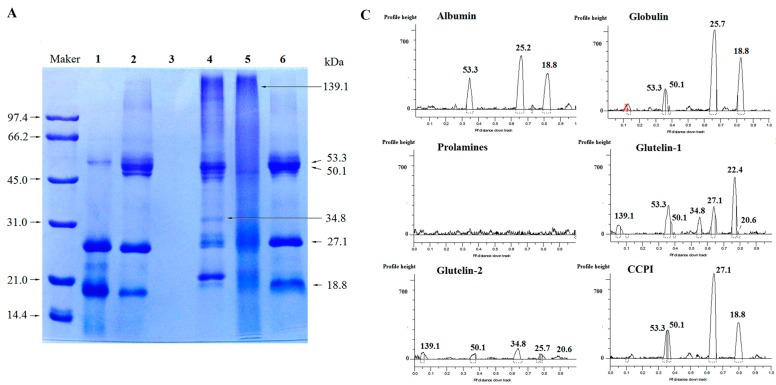

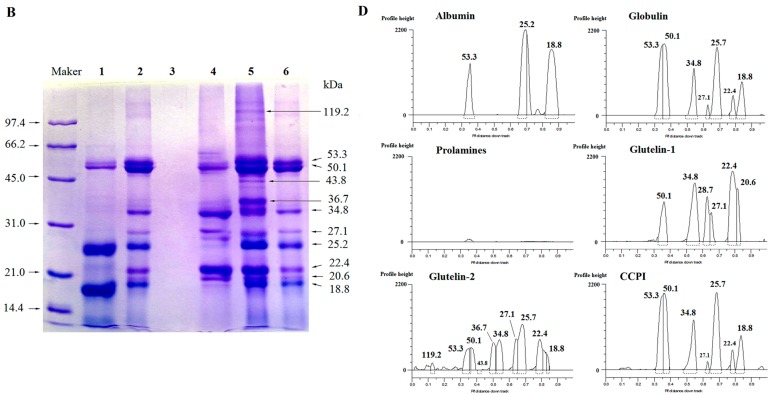
SDS-PAGE patterns and molecular weight distribution profiles of coconut cake protein fractions in the absence (**A**,**C**) or in the presence (**B**,**D**) of β-mercaptoethanol. Maker: molecular weight standards; Track 1: albumin, 2: globulin, 3: prolamine, 4: glutelin-1 and 5: glutelins-2; 6: coconut cake protein isolates (CCPI).

Size heterogeneity among the fractions was revealed by the electrophoretic separations (Figure 1A–D). The globulin showed three main polypeptides under non-reducing conditions (Figure 1A), but seven main polypeptides under reducing conditions (Figure 1B), meaning that disulfide bonds among the polypeptides are present. Kwon et al. [4] also reported that coconut globulin was a hexamer with two disulfide bonds between its acidic polypeptides and basic polypeptides. Moreover, significant difference in molecular weight (Mw) distribution between unreduced and reduced glutelin-1 was observed; the same behavior was found for the glutelin-2 and coconut cake protein isolates (CCPI). This indicated that they consisted of polypeptides linked via one or more disulfide bonds. In contrast, the albumin showed three main polypeptides in both non-reducing and reducing conditions (Figure 1A,B), indicating that there was no disulfide bond in albumin. The components of prolamines were too faint to detect this behavior.

### 2.3. Amino Acid Composition

As shown in Table 1, all the fractions except albumin had a higher TEAA (total essential amino acid) than that recommended by the FAO/WHO [8], and the in vitro protein digestibility (IVPD) of albumin, globulin and glutelin-2 was much higher than that of soybean protein (71.59%) and oil palm kernel protein (68.20%) [9,10], indicating that these fractions had good nutritional qualities. Furthermore, both the globulin and prolamine exhibited a high content of aromatic amino acid (threonine and phenylalanine) and sulfur amino acid (methionine). The globulin also showed relatively high NPS (the frequency of nonpolar side chains) and HФ (the average hydrophobicity), indicating its high content of hydrophobic amino acids. It was reported that aromatic, sulfur and hydrophobic amino acid residues of proteins all contributed to the antioxidant activity [1].

### 2.4. Antioxidant Activities

#### 2.4.1. Free Radical Scavenging Activity

Hydroxyl radical is one of the most reactive radicals, and it can attack almost every molecule in living cells, while superoxide anion radical is a potential precursor of stronger reactive oxidative species such as hydroxyl radical [11]. Moreover, ABTS radical scavenging activity is employed to estimate the total antioxidant activity of antioxidants [12]. Compared to BHT (butylated hydroxytoluene, 100 µg·mL^−1^), the prolamine, glutelin-1 and glutelin-2 had a higher ABTS radical scavenging activity (Table 2), which is probably attributable to the high content of aromatic amino acids (Table 1), which could serve as hydrogen donors and inhibit the radical-mediated peroxidizing chain reaction [2,13]. The highest ·OH scavenging activity was found on prolamine and glutelin-2, perhaps due to the high NPS and HФ (Table 1). Increased hydrophobicity of proteins enhances the antioxidant activity, as it allows the peptide to reach hydrophobic targets [1]. Moreover, prolamine and glutelin-1 also showed a higher superoxide radical scavenging activity than BHT, perhaps resulting from the high content of histidine (Table 1), in which the imidazole group had proton-donation ability [2,11,14]. The results indicated that the prolamine, glutelin-1 and glutelin-2 had high free radical-scavenging activity.

#### 2.4.2. Chelating Activity and Reducing Power

The results in Table 2 also demonstrate that the globulin and prolamine showed excellent chelating ability, which was much higher than that of BHT and hazelnut protein (74.0%), which is probably attributable to the high content of sulfur amino acids (Table 1) [1,15]. Moreover, all the fractions—especially prolamine—showed higher reducing power than BHT (Table 2), which may be a result of the high NPS and high content of aromatic amino acids [2].

#### 2.4.3. DNA Damage Protection

**Figure 2:**
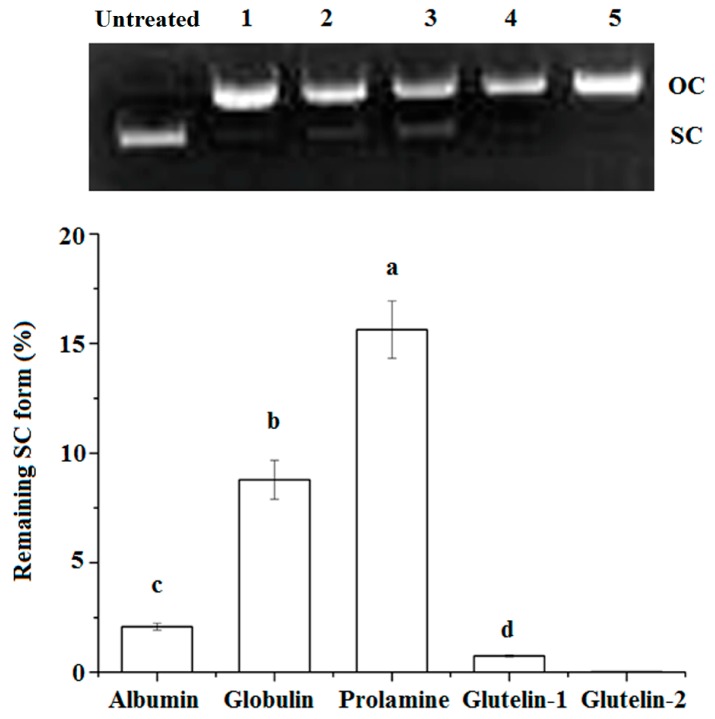
Protective effects of coconut cake protein fractions against DNA damage at 2 mg·mL concentration. SC, super-coiled form; OC, open-circular form; Different small letters a–d on the bar indicate significant difference (*p* < 0.05) in the comparison between samples.

The super coiled (SC) form of pBR322 plasmid DNA will be transformed to its open–circular (OC) form after oxidative damage, and some antioxidants can protect the SC from degradation [16]. As shown in Figure 2, both prolamine and globulin showed protection against DNA damage, in accordance with their high chelating ability on Fe^2+^ and their high radical scavenging activity (Table 2). A previous study has also demonstrated that antioxidants could protect DNA from oxidative damage via chelating Fe^2+^ ion or scavenging the radical produced during the oxidation reaction [11].

### 2.5. LC-MS/MS Analysis

The reported results in Table 2 and Figure 2 demonstrated that the globulin and glutelin-2 had relatively high antioxidation properties. In order to identify which protein segment is endowed with the antioxidant properties, the globulin and the glutelin-2 fractions were subjected to LC-MS/MS (liquid chromatography–mass spectrometry) analysis after tryptic cleavage, and the results are shown in Table 3.

As shown in Table 3, eight and twelve peptides were identified from globulin and glutelin-2, respectively. These peptides were in a molecular weight (Mw) range of 610.45–1057.58 Da, and those containing 5–8 amino acids were predominant. Peptides RPFNLFHK, LPILR, VIEPR, VVLYR and ADVFNPR isolated from the globulin and glutelin-2 were also obtained from palm kernel proteins, and they exhibited high superoxide radical scavenging activity and can effectively protect vascular cells against H_2_O_2_ induced damage [17,18]. Moreover, a previous study identified the peptide ADVFNPR from palm kernel glutelin-1 and found it to have high scavenging ability on hydroxyl radical (IC_50_: 22.16 mg·mL^−1^) [19]. This also contributed to the antioxidation of the globulin and glutelin-2. Furthermore, amino acid residues such as histidine, proline, valine, arginine and aromatic amino acid could be associated with the antioxidant activity of antioxidant peptides [20], which is consistent with the result of this study.

## 3. Materials and Methods

### 3.1. Materials

Coconut cake was purchased from Nanye Coconut Food Co., Haikou, China. BHT, ABTS (2,2′-azino-bis (3-ethylbenzothiazoline-6-sulphonic acid)), 2-Deoxy-d-ribose and plasmid pBR322 DNA were purchased from Shanghai Yeyuan Biotech. Co., Ltd., Shanghai, China. Low molecular weight protein markers were purchased from Shanghai DINGUO Biotech. Co., Ltd., Shanghai, China. Other reagents were all of analytical grade.

### 3.2. Fractionation of CCP Fractions

**Figure 3:**
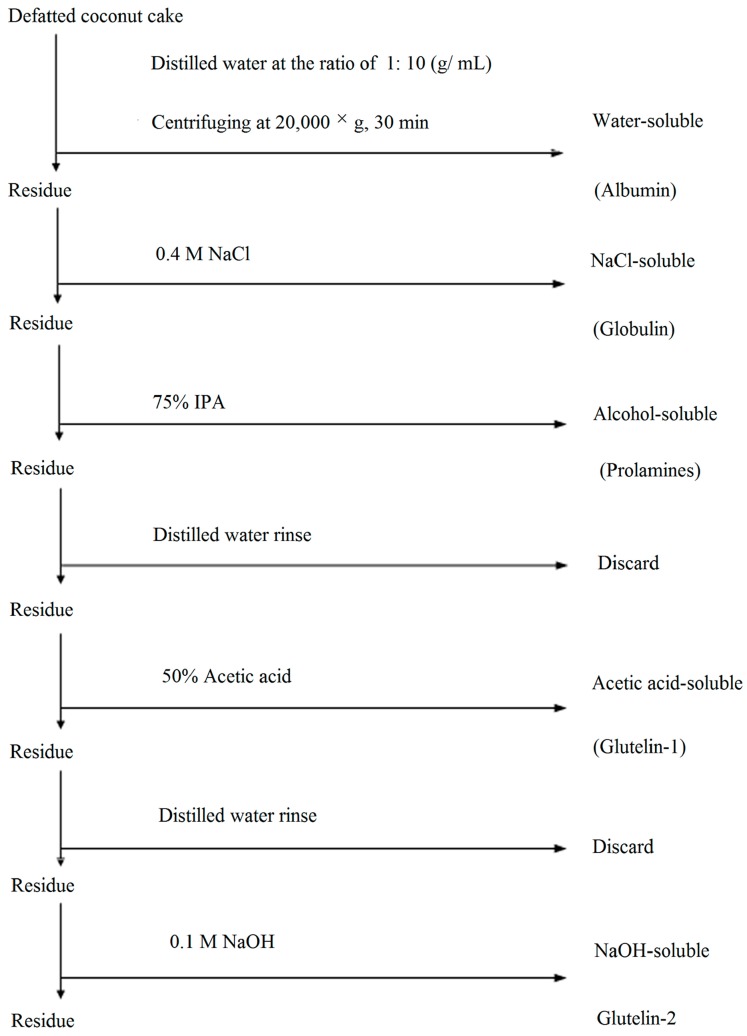
Flow sheet of the protocol used for fractionation of coconut cake protein by different solubility.

Prior the sequential fractionation described by Kwon et al. [4], the coconut cake was defatted three times with *n*-hexane (1:10, *w*/*v*), dried, ground and passed through a sieve of 0.2 mm mesh to obtain defatted coconut cake (DCC). Different solvents, including 0.4 mol·L^−1^ NaCl, 70% (*v*/*v*) 2-propanol (IPA), 50% (*v*/*v*) glacial acetic acid and 0.1 mol·L^−1^ NaOH, were used in sequence to extract the five fractions, and protocol was summarized in Figure 3. Correspondingly, water-, NaCl-, IPA-, acetic acid- and NaOH-soluble proteins were designated as albumin, globulin, prolamine, glutein-1 and glutein-2, respectively. Each fraction was extracted with a ratio of 1:10 (g·mL^−1^) after 8 h at 4 °C. After centrifugation at 20,000× *g* for 30 min, the supernatant was collected and dialyzed against deionized water at 4 °C for 48 h, and then lyophilized in a freeze dryer (LABCONCO 2.5, Kansas, MO, USA). Extraction with each solvent was carried out in triplicate, and all the resulting fractions were stored at −20 °C. Protein concentration of each fraction was determined by the Bradford method [21]. Coconut cake protein isolate (CCPI) was prepared following the method of Angelia et al. [5].

### 3.3. Sodium Dodecyl Sulfate-Polyacrylamide Gel Electrophoresis (SDS-PAGE)

SDS-PAGE was performed according to the method of Laemmli [22] on 7.5% stacking gel and 12% separating gel. To analyze the protein fractions under reducing conditions, β-mercaptoethanol was added to the sample buffer. Molecular weight of the subunits were estimated using these markers: phosphorylase *b* (97.4 kDa), BSA (66.2 kDa), ovalbumin (45.0 kDa), carbonic anhydrase (31.0 kDa), trypsin inhibitor (20.1 kDa) and lysozyme (14.4 kDa).

### 3.4. Amino Acid Composition

Amino acid composition (expressed as g/100 g protein) of each CCP fraction was determined by the method of Wang et al. [23] with an automatic amino acid analyzer (HITACHI L-8900, Tokyo, Japan). The Bigelow parameters were also calculated, including NPS (the frequency of nonpolar side chains), HФ (the average hydrophobicity) and P (ratio of polar to nonpolar side chains). Where, NPS was calculated by counting the Trp, Ile, Tyr, Phe, Pro, Leu and Val residues and expressing the sum as a fraction of the total number of residues [1]. In vitro protein digestibility (IVPD) was determined following the method of Saunders et al. [24]. 0.5 g protein was suspended in 10 mL of 0.1 M HCI and mixed with 25 mg pepsin (5 × 10^4^ U/g),and gently shaken at 37 °C for 48 h and then centrifuged at 10,000 × *g* 30 min. The supernatant and the precipitate were collected, respectively. The precipitate was suspended in 10 mL of 0.1 M sodium phosphate buffer (pH 8.0) and 2.5 mg trypsin (1 × 10^5^ U/g) was added. The mixture was gently shaken at 23 °C for 16 h. The digested mixture was then centrifuged and trichloroacetic acid (TCA) was added to the supernatant to reach a concentration of 8 M in the solution. The supernatant previously obtained from pepsin digestion was also treated in a similar manner. Precipitated proteins were removed by centrifugation at 10,000 × *g* 25 min. The TCA-soluble nitrogen content of the supernatant was determined by Branford method [21]. IVPD was expressed as percentage of enzymatic digestion.

### 3.5. Antioxidant Activities

#### 3.5.1. ABTS Radical Scavenging Activity

Following the method of Arts et al. [12], 20 µL of protein solution (100 µg·mL^−1^) was mixed with 2 mL of ABTS (2,2′-azino-bis (3-ethylbenzothiazoline-6-sulphonic acid)) solution (7 mM), and then incubated in the dark at 30 °C for 6 min. Then, absorbance at 734 nm was recorded. BHT (100 µg·mL^−1^) was used as the control. The activity was calculated as follows:

(1) $$\mathrm{Scavenging}\;\mathrm{activity}\left(\%\right)=[1-(A_{S}-A_{B})/A_{C}]\times 100$$

where A_C_ was the absorbance of the control (distilled water replaced samples), A_B_ was the absorbance of blank (without ABTS) and A_S_ was the absorbance of the mixture containing samples.

#### 3.5.2. Hydroxyl Radical Scavenging Activity

Following the method of Ren et al. [14], the reaction mixture contained 100 µL sample solution (100 µg·mL^−1^), 100 µL FeSO_4_-EDTA (10 mM), 100 µL 2-deoxyribose (10 mM), 1.4 mL phosphate buffer (pH7.4, 0.1 M) and 100 µL H_2_O_2_ (10 mM). After incubation at 37 °C for 1 h, the reaction solution was mixed with 1.0 mL trichloroacetic acid (28 g·L^−1^) and 1.0 mL thiobarbituric acid (10 g·L^−1^), and then incubated at 100 °C for 20 min. The absorbance was measured at 532 nm. The activity was determined as follows:

(2) $$\mathrm{OH}\;\mathrm{scavenging}\;\mathrm{activity}\;\left(\%\right)=[1-(A_{S}-A_{B})/A_{C}]\times 100$$

where A_B_ was the absorbance of blank (without 2-deoxyribose).

#### 3.5.3. Superoxide Radical-Scavenging Activity

Superoxide radical-scavenging activity was measured using the pyrogallol assay as described by Gao et al. [25]. 0.1 mL of sample solution was mixed with 3 mL of pyrogallol solution (3 mM), the absorbance at 320 nm was recorded at 30 s intervals for 8 min. The scavenging activity was calculated from the absorbance at 320 nm in the presence or absence of samples.

#### 3.5.4. Metal Chelating Capacity

Following the method of Jeong et al. [16], the mixture of protein (0.45 mL, 100 µg·mL^−1^) and FeCl_2_ (45 µL, 2 mM) was reacted with ferrozine (90 µL, 5 mM) for 30 min. Then the absorbance was read at 562 nm, and chelating activity was calculated as follows:

(3) $$\mathrm{Chelating}\;\mathrm{activity}\;\left(\%\right)=[1-(A_{S}-A_{B})/A_{C}]\times 100$$

where A_B_ was the absorbance of the blank (without ferrozine).

#### 3.5.5. Reducing Power

Reducing power was determined by the method of Yen et al. [26]. Aliquot of sample solution (100 µg·mL^−1^) was mixed with 0.2 M phosphate buffer (pH 6.6) and potassium ferricyanide (0.01 g·mL^−1^). The mixture was first incubated at 50 °C for 20 min. An aliquot (2.5 mL) of TCA (0.1 g·mL^−1^) was added to the mixture, followed by centrifugation at 3000× *g* for 10 min. The supernatant (2.5 mL) was mixed with 2.5 mL of distilled water and 2.5 mL of ferric chloride (1 g·mL^−1^), and the absorbance at 700 nm was read after 10 min. BHT (100 µg·mL^−1^) was used as the positive control. Increased absorbance at 700 nm of the reaction mixture indicated increased reducing power.

#### 3.5.6. Protection on DNA from Oxidative Damage

A 5 µL of sample solution (2 mg·mL^−1^) was added to 11 µL of the reaction mixture, which contained 6 mM H_2_O_2_, 1.5 mM FeSO_4_ and 0.5 µg of plasmid pBR322 DNA. The mixture was incubated at 37 °C for 1 h [16]. Then electrophoresis was performed in 1% agarose gel. The remaining super-coiled (SC) form against oxidative DNA cleavage was quantified using a gel imaging system (SyneneBOXF3, Cambridge, UK).

### 3.6. LC-MS/MS Analysis

The CCP fractions with high antioxidation and potential antihypertension were subjected to LC-MS/MS analysis. Aliquot of sample solution (25 µL) was mixed with 5 µL of 0.2 M NH_4_HCO_3_ and 3.3 µL DL-Dithiothreitol. After being incubated at 56 °C for 1 h, 3.9 µL of 0.55 M iodoacetamide was added and further incubated at 25 °C for 40 min in the dark. Then 1 µg trypsin was added and incubated at 37 °C for 14 h. Amino acid sequences of the hydrolysates were determined by LC-MS/MS with a coupled Eksigent Nano LC (Eksigent Technologies, Dublin, CA, USA) and Thermo LTQ linear ion trap mass spectrometer (Thermo Fisher, San Jose, CA, USA). The acquired MS/MS data were interpreted using the bioinformatics search engine Mascot version 2.1.0 (Matrix Sciences, London, UK).

### 3.7. Statistical Analysis

Data were subjected to analysis of variance and Duncan value with a confidence interval of 95% was calculated to compare means.

## 4. Conclusions

The albumin, globulin, prolamine, glutelin-1 and glutelin-2 protein fractions were successfully obtained by the sequential extraction method from coconut cake. All these fractions except albumin exhibited higher radical-scavenging activity and ion chelating ability. These fractions except glutelin-2 can also protect DNA from oxidative damage. These results indicate that these fractions can be used as natural antioxidant or food ingredient for some food such as meat patties to prolong shelf life of product. However, whether these fractions have antioxidant properties in vivo will need to be determined through further work.

## Figures and Tables

**Table 1:** Amino acid composition (g/100 g protein), Bigelow parameters and nutritional quality of coconut cake protein fractions and coconut cake protein isolates (CCPI).

Amino Acid Residue	Fractions						FAO/WHO
	Albumin	Globulin	Prolamine	Glutelin-1	Glutelin-2	CCPI	
Essential amino acid							
Isoleucine	2.85 ± 0.22 ^e^	4.12 ± 0.52 ^b^	5.15 ± 0.34 ^a^	3.72 ± 0.23 ^c^	3.22 ± 0.30 ^d^	4.33 ± 1.70 ^b^	3.4
Leucine	4.12 ± 0.36 ^c^	6.51 ± 0.15 ^b^	9.55 ± 0.12 ^a^	6.51 ± 0.17 ^b^	6.29 ± 0.16 ^b,c^	6.83 ± 0.78 ^b^	3.5
Lysine	5.1 5 ± 0.43 ^b^	3.53 ± 0.26 ^d^	8.36 ± 0.39 ^a^	3.52 ± 0.30 ^d^	5.54 ± 0.16 ^b^	4.96 ± 0.65 ^c^	5.8
Methionine	1.26 ± 0.25 ^c^	2.82 ± 0.13 ^a^	2.95 ± 0.53 ^a^	2.14 ± 0.31 ^b^	1.26 ± 0.06 ^c^	1.57 ± 0.45 ^c^	
Phenylalanine	3.05 ± 0.34 ^d^	5.86 ±0 .12 ^a^	5.44 ± 1.54 ^a^	4.6 5± 0.29 ^b^	4.45 ± 0.21 ^c^	4.8 0 ± 0.51 ^b^	
Threonine	3.05 ± 0.35 ^a^	3.21 ± 0.18 ^a^	3.14 ± 0.33 ^a^	3.23 ± 0.04 ^a^	3.00 ± 0.11 ^a^	2.73 ± 0.36 ^b^	2.5
Tryptophan	ND ^l^	ND ^l^	ND ^l^	ND ^l^	ND ^l^	ND ^l^	2.8
Valine	3.72 ± 0.33 ^d^	7.41 ± 0.07 ^a^	5.95 ± 0.61 ^b^	4.35 ± 0.57 ^c^	4.05 ± 0.08 ^c^	4.80 ± 1.02 ^c^	6.6
Histidine	1.74 ± 0.10 ^c^	2.15 ± 0.35 ^b^	2.64 ± 0.48 ^a^	2.54 ± 0.80 ^a^	1.89 ± 0.11 ^c^	2.04 ± 0.45 ^b^	1.9
Tyrosine	3.35 ± 0.47 ^b^	3.64 ± 0.10 ^b^	5.54 ± 0.59 ^a^	5.68 ± 0.86 ^a^	3.59 ± 0.72 ^b^	3.04 ± 1.51 ^c^	0.5
Nonessential amino acid							
Asparagine	6.95 ± 0.49 ^b^	8.51 ± 0.42 ^a^	4.14 ± 0.10 ^d^	5.7 5± 0.59 ^c^	8.31 ± 0.50 ^a^	8.82 ± 0.71 ^a^	
Serine	3.45 ± 0.40 ^d^	4.55 ± 0.51 ^b^	5.61 ± 0.31 ^a^	3.89 ± 0.34 ^c^	3.84 ± 0.06 ^c,d^	4.63 ± 1.08 ^b^	
Glutamic acid	23.8 ± 1.56 ^b^	20.7 ± 4.52 ^b^	1.92 ± 0.42 ^d^	17.01 ± 3.61 ^c^	27.21 ± 0.88 ^a^	24.50 ± 3.47 ^b^	
Glycine	4.01 ± 0.42 ^b^	4.78 ± 0.14 ^a^	2.13 ± 0.57 ^c^	4.54 ± 0.07	4.95 ± 0.31 ^a^	4.90 ± 0.28 ^a^	
Alanine	2.84 ± 0.24 ^d^	3.89 ± 0.23 ^c^	9.21 ± 1.21 ^a^	3.93 ± 0.45 ^b^	3.74 ± 0.35 ^c^	4.06 ± 0.90 ^b^	
Arginine	17.65±0.0.36 ^a^	15.15± 0.23 ^b,c^	3.64 ± 0.18 ^c^	14.25± 1.37 ^b,c^	16.11 ± 1.27 ^a^	13.62± 3.27 ^b,c^	
Proline	2.75 ± 0.18 ^c^	3.14 ± 0.23 ^b,c^	4.21 ± 0.42 ^a^	3.22 ± 0.39 ^b,c^	2.45 ± 0.82 ^d^	3.60 ± 0.50 ^b^	
Bigelow parameters							
NPS ^f^	0.22 ± 0.03 ^d^	0.31 ± 0.06 ^b^	0.45 ± 0.04 ^a^	0.32 ± 0.03 ^b^	0.24 ± 0.11 ^d^	0.28 ± 0.05 ^c^	
P ^g^	2.79 ± 0.44 ^a^	1.63 ± 0.03 ^b^	0.58 ± 67 ^c^	1.53 ± 0.16 ^b,c^	2.46 ± 0.13 ^a,b^	1.97 ± 0.31 ^b^	
HФ ^h^	844.00 ^e^	1100.01 ^a^	1013.29 ^c^	990.21 ^d^	1111.65 ^a^	1084.66 ^b^	
Nutritional quality							
TEAA ^i^	28.29 ± 1.19 ^c^	39.25 ± 0.95 ^b^	48.72 ± 2.30 ^a^	36.34 ± 1.41 ^b^	33.29±1.12 ^b,c^	35.10 ± 3.15 ^b^	12.7
E/T ^j^ (%)	31.52 ^d^	39.26 ^c^	61.22 ^a^	40.86 ^b^	33.32 ^d^	35.37 ^c^	
IVPD ^k^ (g/100 g)	90.31 ± 3.48 ^a^	88.26 ± 2.58 ^b^	ND ^l^	72.74 ± 4.11 ^c,d^	76.35 ± 5.12 ^c^	86.97 ± 1.62 ^a^	

^a^^–e^ Different superscripts indicate significant difference (*p* < 0.05) in the comparison of samples in the same row; ^f^ The frequency of nonpolar side chains; ^g^ Ratio of polar to nonpolar side chains; ^h^ The average hydrophobicity; ^i^ Total of essential amino acids; ^j^ Ratio of essential amino acid to total amino acids; ^k^ In vitro digestibility; ^l^ ND, Not detected.

**Table 2:** Radical scavenging ability and antioxidant activity of coconut cake protein fraction at concentration of 100 µg·mL^−1^.

Fractions	Free Radical Scavenging Ability (%) ^a^			Antioxidant Activity	
	ABTS Radical	Hydroxyl Radical	Superoxide Radical	Chelating Activity (%)	Reducing Power
Albumin	5.04 ± 0.59 ^d,e^	8.83 ± 0.98 ^f^	14.99 ± 2.10 ^e^	5.13 ± 1.44 ^e,f^	0.121 ± 0.010 ^c^
Globulin	2.07 ± 0.64 ^e^	16.87 ± 1.46 ^e^	10.91 ± 0.79 ^e^	89.14 ± 3.42 ^b^	0.016 ± 0.005 ^e^
Prolamine	66.05 ± 1.17 ^b^	68.27 ± 6.52 ^c^	56.31 ± 2.60 ^c^	80.38 ± 6.11 ^c^	0.156 ± 0.003 ^b^
Glutelin-1	52.58 ± 0.22 ^c^	20.48 ± 1.85 ^d^	67.82 ± 2.72 ^b^	3.60 ± 0.48 ^f^	0.138 ± 0.003 ^b^
Glutelin-2	53.76 ± 0.25 ^c^	68.27 ±2.55 ^c^	4.14 ± 0.75 ^f^	5.02 ± 1.17 ^e,f^	0.100 ± 0.003 ^d^
BHT ^g^	5.81 ± 1.46 ^d,e^	77.54 ± 3.45 ^b^	48.68 ± 0.83 ^d^	41.45 ± 0.78 ^d^	0.002 ± 0.000 ^f^

^a^ Different superscripts ^b–f^ in the same column indicate significant difference (*p* < 0.05) in the comparison between samples; ^g^ BHT was used as the comparison.

**Table 3:** Peptides from coconut cake protein fractions isolated and identified by LC-MS/MS and their potential biological activity.

Fraction	Peptide Sequence ^a^	Molecular Weight (Da)	Possible Biological Activities	References
Globulin	NFLEK	649.34		
	ATHELR	725.38		
	EDKLER	788.4		
	AGTIVSFANR	1034.55	Antioxidative	[17]
	SWPFGESR	964.44		
	RPFNLFHK	1057.58	Antioxidative	[17]
	GREEEEGR	960.43		
	TWLAGR	702.38		
Glutelin-2	VIEPR	612.36	Antioxidative	[18]
	RVIEPR	768.46	Antioxidative	[17]
	QFLLAGR	803.46		
	ENILR	643.36		
	KLQCR	703.38		
	IKQNIGDPR	1039.58		
	QNIGDPR	798.40		
	RADVFNPR	973.51	Antioxidative	[17]
	ADVFNPR	817.41	Antioxidative	[17,19]
	ITTLNSEK	904.49		
	LPILR	610.45	Antioxidative	[18]
	VVLYR	648.40	Antioxidative	[18]

^a^ From National Center for Biotechnology Information (NCBI).
